# B7H4 Role in Solid Cancers: A Review of the Literature

**DOI:** 10.3390/cancers16142519

**Published:** 2024-07-11

**Authors:** Miriam Dawidowicz, Anna Kot, Sylwia Mielcarska, Katarzyna Psykała, Agnieszka Kula, Dariusz Waniczek, Elżbieta Świętochowska

**Affiliations:** 1 Department of Oncological Surgery, Faculty of Medical Sciences in Zabrze, Medical University of Silesia, 41-808 Katowice, Poland; 2 Department of Medical and Molecular Biology, Faculty of Medical Sciences in Zabrze, Medical University of Silesia, 19 Jordana, 41-800 Zabrze, Poland

**Keywords:** B7H4, immunotherapy, immune checkpoint, B7 family, immunological tumour microenvironment

## Abstract

**Simple Summary:**

B7H4 emerges as a promising therapeutic target exhibiting negative costimulatory activity and whose expression is aberrant in a wide range of solid tumours. This molecule has obtained increased attention as the immune checkpoint is highly expressed in immune “cold” tumours in which the presence of PD-1 and PD-L1- is minimal, making these tumours unresponsive to most currently used immunotherapies. Deficient expression of B7H4 in normal tissue and its overexpression in malignant neoplasms makes B7H4 an attractive immunotherapy agent with potentially lower toxicity than anti-PD-1 and PD-L1 treatment. Numerous clinical trials have evaluated B7H4 targeting immunotherapy using various treatment modalities, including monoclonal antibodies, bispecific antibodies, antibody-drug conjugates, and CAR T cells. In this review, we aimed to update the current state of knowledge regarding B7H4’s role in tumour promotion and immune evasion and summarise results from clinical trials assessing immunotherapies targeting B7H4 in solid tumours.

**Abstract:**

Anti-cancer immunotherapies entirely changed the therapeutic approach to oncological patients. However, despite the undeniable success of anti-PD-1, PD-L1, and CTLA-4 antibody treatments, their effectiveness is limited either by certain types of malignancies or by the arising problem of cancer resistance. B7H4 (aliases B7x, B7H4, B7S1, VTCN1) is a member of a B7 immune checkpoint family with a distinct expression pattern from classical immune checkpoint pathways. The growing amount of research results seem to support the thesis that B7H4 might be a very potent therapeutic target. B7H4 was demonstrated to promote tumour progression in immune “cold” tumours by promoting migration, proliferation of tumour cells, and cancer stem cell persistence. B7H4 suppresses T cell effector functions, including inflammatory cytokine production, cytolytic activity, proliferation of T cells, and promoting the polarisation of naïve CD4 T cells into induced Tregs. This review aimed to summarise the available information about B7H4, focusing in particular on clinical implications, immunological mechanisms, potential strategies for malignancy treatment, and ongoing clinical trials.

## 1. Introduction

B7H4 is a promising target for novel immunotherapies in many cancers and autoimmune diseases. B7H4 (B7 homolog 4, aliases B7x, B7H4, B7S1, VTCN1) is a seventh member of the B7 family of cell signalling ligands discovered in 2003. It acts as an immunosuppressant that regulates the innate and adaptive immune systems. Overexpression of B7H4 in many cancers has been associated with the activation or suppression of multiple pathways that ultimately enable the tumour’s evasion from immune system surveillance [1,2,3].

B7H4 was identified with known B7 family members in 2003 by three separate laboratories [1,3,4]. Its gene name is V-set domain containing T cell activation inhibitor 1 (*VTCN1*) and is located on 1p11.1, consisting of six exons and five introns. Mature VTCN1 protein consists of 282 amino acids, constructing one signal peptide, two extracellular immunoglobulins (IgV1 and IgV2) domains, one transmembrane domain, and a small cytoplasmic tail [2].

*VTCN1* transcripts have been reported in various tissues, including the placenta, kidney, liver, spleen, ovary, and testis [1,3,4]. In contrast to constitutive expression of the *VTCN1* mRNA, no staining in immunohistochemistry analyses was found in healthy tissues such as the lung, colon, liver, skeletal muscle, kidney, pancreas, small bowel, and breast. These results suggest the posttranslational regulation of *VTCN1* expression [2]. B7H4 is expressed in the physiological state at the protein level on T cells, B cells, monocytes, and dendritic cells (DCs) [3].

On the other hand, B7H4 overexpression has been confirmed in many cancerous tissues, especially in non-inflamed (cold) immune contexture cancers [5,6,7]. The mechanism of B7H4 expression regulation is poorly known. It is likely upregulated by some cytokines, such as IL-10 or IL-6. This way, Tregs could stimulate macrophages to secrete IL-10 and IL-6, which would lead to the inhibition of T-cell proliferation. Although numerous factors have been detected to influence B7H4 expression in vitro, the mechanism behind B7H4 upregulation in different types of immune and cancer cells remains to be elucidated.

In this review, we provide an overview of the current state of knowledge regarding the immunological function of B7H4 in solid cancer. We then focus on the therapeutic approach to targeting B7H4 and its potential as a therapeutic target in immunotherapy.

## 2. B7H4 in Immunity and Autoimmunological Diseases

B7H4 exerts its inhibitory function on the immune system by its suppressive effect on CD4 and CD8 T cells. In vitro studies demonstrated that the activity of B7H4 leads to the suppression of T cell effector functions, including inflammatory cytokine production, cytolytic activity, and inhibition of the proliferation of T cells by arresting their progression through the cell cycle at the G0/G1 phase [1,2,3]. B7H4 plays a role in shaping the immunosuppressive environment. It promotes the activity of natural regulatory T cells (Treg) and modulates the polarisation of naïve CD4 T cells into induced Tregs, concurrently inhibiting conversion to the inflammatory TH1 and TH17 subtypes [8].

**Figure 1:**
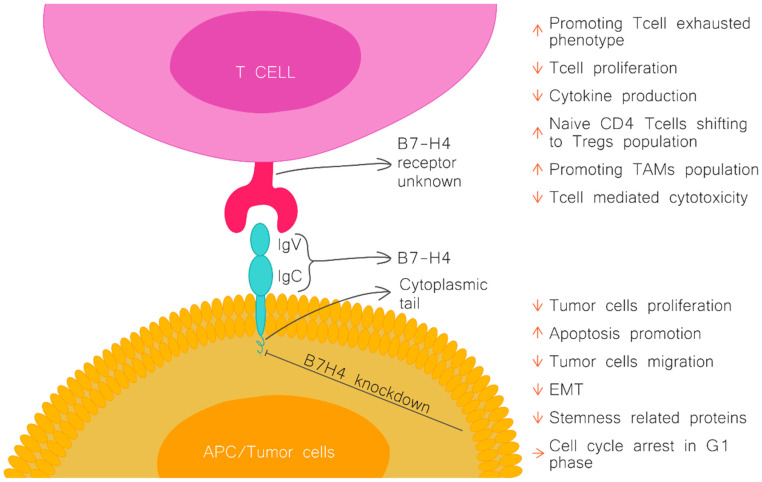
B7H4 structure and function. Figure upper part, the effect of B7H4 interaction with its receptor (unknown). Figure lower part, the effect of B7H4 knockdown on tumour cells. APC-antigen presenting cells; Treg, regulator T cells; TAMs, tumour associated macrophages; EMT, epithelial-to-mesenchymal transition.

In addition to impacting adoptive immunity, there is evidence that B7H4 also regulates innate immunity. B7H4 has been demonstrated to inhibit the production of neutrophils from bone marrow progenitors, and another study showed that B7H4 binds to tumour-infiltrating neutrophils through an unknown receptor (Figure 1) [9,10].

An impaired immune response, mediated by B7H4, may result not only in the progression of cancer diseases but also in autoimmune diseases. Contrary to cancers, B7H4 expression and function in autoimmune disorders have been reported to be decreased, e.g., in rheumatoid arthritis, type 1 diabetes (T1D), and juvenile idiopathic arthritis in humans [11,12,13]. In detail, the studies showed that B7H4 expression on pancreatic islet cells inhibits CD4 and CD8 T cell-mediated autoimmunity and thus leads to preventing diabetes progression [14].

## 3. B7H4 Expression in Solid Cancers

Tumour cells evade T cell-mediated immunity by exploiting the inhibitory functions of B7H4. B7H4 expressed on both tumour and host cells reduces the activation and subsequent effector functions of tumour-infiltrating CD4 and CD8 T cells, such as inflammatory cytokine production and cytolytic activity. Furthermore, B7H4 induces the shifting of effector T cells into an “exhausted” T cell phenotype marked by the co-expression of PD-1 and Tim-3 [10,15,16]. The B7H4 immune checkpoint also plays a pivotal role in shaping an immunosuppressive environment in cancers by promoting immunosuppressive cells, including Tregs, myeloid-derived suppressor cells, and macrophages [10]. Moreover, tumour-associated macrophages (TAMs) can express B7H4 in many cancers. TAMs are a significant immunosuppressive cell population in many tumour types [15,17,18]. B7H4+ TAMs, but not tumour cells, correlate with a worse patient prognosis in ovarian cancer and, similarly, in hepatocellular carcinoma [15,17,19]. Another substantial population of cells in the immune tumour microenvironment (iTME) with positive expression of B7H4 are cancer stem cells (CSCs) [20]. B7H4 contributes to the maintenance of the CSC population preferentially localised within the border of the tumour [21,22] and contributes to cancer resistance to immune checkpoint inhibitor (ICI) therapies [22].

**Figure 2:**
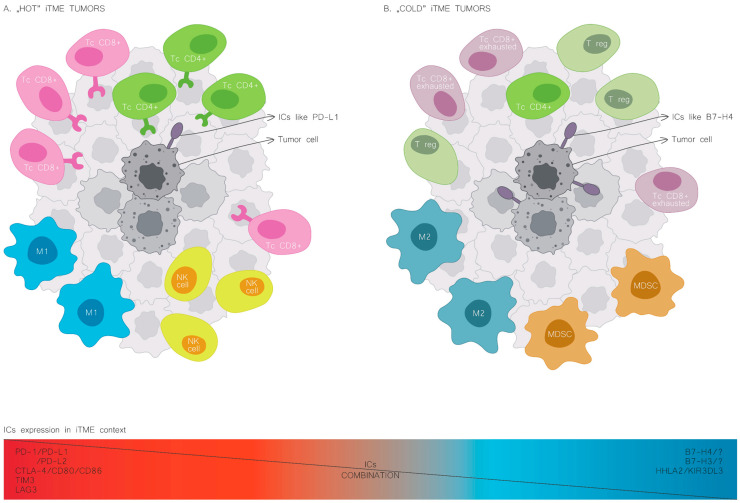
Cold and hot immune tumour microenvironment. The leading cellular players shaping iTME composition in the hot tumour phenotype (**A**) and the cold tumour phenotype (**B**). NK, natural killer cells; M1, macrophages of type 1; M2, macrophages of type 2; MDSC, myeloid-derived suppressor cells; Tc CD8, T cells CD8 lymphocytes; Tc CD4, T cells CD4 lymphocytes; T reg, regulator T cells; PD-L1, programmed cell death-ligand 1; B7H4, B7 homolog 4.

The expression patterns of immune checkpoints in cancers mark a distinct iTME. B7H4 expression occurs more frequently in human cancers with an immunologically “cold” microenvironment, characterised by decreased immune cell infiltration [6,23]. Moreover, B7H4 is often co-expressed with different immune checkpoints, such as B7H3 and HHLA2 [24,25]. On the contrary, PD-L1 expression correlates with increased immune cell infiltration, and the co-expression of PD-L1 and B7H4 rarely occurs (Figure 2) [26,27].

In haematologic malignancies, the role of B7H4 may differ from that of solid cancer. However, the data are limited. In acute myeloid leukaemia (AML), B7H4 is expressed on leukaemia-initiating cells and enriched CD34+ AML cells. In silico analysis from the Leukemia Gene Atlas showed that B7H4 expression level was positively correlated with the overall survival of AML patients [28]. Similarly to the PD-1 pathway in T cell lymphoma, where PD-1 acts as a tumour suppressor, the B7H4 might also play a suppressive role in AML [29].

### 3.1. Breast Cancer

The upregulation of B7H4 expression in breast cancer (BC) has been confirmed at the mRNA and protein levels. IHC results revealed intensive staining for B7H4 in the cell membrane and cytoplasm of cancer cells with weak staining in adjacent normal tissue [30]. High B7H4 expression was related to a high Ki67 index, more advanced TNM stage, and decreased overall survival of patients [31]. Co-expression of B7H4 with other immune checkpoints, such as PD-L1, has been rarely reported [32]. However, the presence of B7H4 is more common in tumours that do not express hormone receptors and correlates negatively with the expression of android receptors. In a study conducted by Sun-Seog Kweon et al., the highest and the lowest percentage of tumours positive for B7H4 expression was found in HR−/HER2+ and HR+/HER2+ cases—respectively, 60% and 25% [30,33].

B7H4 in tumour cells correlated inversely with the number of tumour-infiltrating CD8 T lymphocytes in breast invasive ductal cancer tissues. In a mouse tumour model, B7H4 overexpression on the tumour surface fostered tumour growth in immunocompetent mice by suppressing the activation, expansion, and cytotoxicity of CD8 tumour-specific T cells. However, in further studies, the application of T cells with B7H4-specific chimeric antigen receptors (CARs) showed an association between a loss of B7H4 expression in cancer cells escaping from T cell cytotoxicity and their enhanced epithelial-to-mesenchymal transition (EMT). At the same time, B7H4 upregulation reduced CD8 T cell cytotoxicity against cancer cells and decreased the proliferation and migration of cancer cells. Additionally, there was an association between the downregulation of B7H4 expression and the more advanced TNM stage, suggesting that cancer cells with decreased expression of B7H4 escape from the tumour immune environment and spread to local lymph nodes [34]. These contradictory results indicate that the B7H4 interplay with BC iTME needs further elucidation to entwine the network of B7H4 influences.

### 3.2. Gastrointestinal Cancers

#### 3.2.1. Oesophageal Cancer

Several studies aimed to investigate B7H4 expression in oesophageal squamous cell carcinoma (ESCC), demonstrating its significant upregulation in the cancerous tissue compared with normal tissues [20,35,36,37]. Positive B7H4 immunohistochemical staining was observed in 95.5% of specimens of ESCC tissues; of them, 62% belong to the higher B7H4 expression group [35]. Piao L and colleagues reported similar overexpression. However, the positive staining rate for B7H4 was 53.8% in ESCC tissues [20]. The most prominently B7H4 overexpressing cells were cancer cells, stromal fibroblasts, and macrophages, and B7H4 staining was particularly evident at the cancer cell invasive front and lymphatic invasion cancer cells [20]. In approximately 49.5% of ESCC tissues, the high co-expression of B7H4 and B7H3 occurred, and in another study, the co-expression rate was 71.2% [36].

B7H4 expression was also correlated with the patient’s clinicopathological features. Chen LJ and Piao L demonstrated the association of B7H4 with the patient’s gender, distant metastasis, and TNM stage [20,35,36]. Moreover, higher B7H4 expression correlates with shorter overall survival (OS) [35] and disease-free survival (DFS) [20].

The immunological tumour microenvironment (iTME) appeared influenced by B7H4 expression in ESCC. B7H4 in tumour cells is inversely correlated with CD3 and CD8 TIL densities and positively correlated with the intensity of Foxp3 T lymphocytes, which marks the Tregs population. Furthermore, B7H4 was strongly associated with CD68 macrophages. CD68 marks the tumour-associated macrophages (TAMs), the significant immunosuppressive cell population of the iTME [35].

Cancer stem cells (CSCs) are one of the sources of resistance to ICI therapies, as mentioned above. B7H4 expression in ESCC was significantly correlated and co-localised with stemness-related proteins such as Sox9, LSD1, Oct4, and LGR5, thus indicating some relation between B7H4 and CSCs [20]. Notably, B7H4 expression was positively associated with cyclin D1 and p27 in ESCC. The results revealed that B7H4 can stimulate cell cycle progression through the upregulation of the cell cycle-related proteins cyclin D1 and p27. B7H4 in ESCC tissue was associated with the expression of pPI3K, pAkt-Ser473, and p65 NFκB. The activation of PI3K/Akt/NFκB signalling is essential for the oncogenic effect of B7H4 on cell invasion and the stem cell-like properties of cancer cells. However, the specific mechanism still needs further elucidation [20]. Interestingly, analysis of ESCC cell lines revealed nonimmunological effects of B7H4 expression. B7H4 knockdown inhibited cell growth in the Eca-109 cell line [37]. In murine cancer models, B7H4 and its function-enhancing oesophageal precancerous lesions have been associated, at least in part, with IL-6/STAT3 activation [38]. Further analysis of this pathway on ESCC cell lines confirmed that B7-H4 silencing dampened IL-6 secretion through JAK2/STAT3 pathway inactivation, accounting for cell proliferation inhibition and apoptosis induction [39].

#### 3.2.2. Gastric Cancer

The overexpression of B7H4 in gastric cancer (GC) has also been evaluated in several studies. The rate of B7H4-positive cancer tissue samples varied from 44.9% to 80% [40,41]. B7H4 also has a soluble form, sB7H4, which can be detected at elevated levels in GC patient’s blood [42]. Guo L et al. analysed the expression of B7H4, B7H3, and PD-L1 in tissues ranging from chronic superficial gastritis and atrophic gastritis samples to low-grade intraepithelial neoplasia samples, high-grade intraepithelial neoplasia samples, and gastric adenocarcinoma [40]. They noticed that the expression of each immune checkpoint gradually rose from chronic gastritis samples to gastric adenocarcinomas; however, in all stages of carcinogenesis, the scores for B7H4 expression were markedly higher than those for PD-L1 and B7-H3 expression [40]. In GC samples, the overexpression of B7H4 was confirmed on tumour cells, tumour-infiltrating immune cells, TAMs, circulating monocytes, and intratumoural neutrophils [40,43,44].

B7H4 expression is also correlated with clinical cancer features such as cancer myometrium invasion, lymphatic invasion, venous invasion, lymph node metastasis, and TNM stage [41,43,45]. B7H4 is associated with worse OS, DFS, and risk of recurrence in GC [42]. The OS was shorter in the B7H4 high-expression group, as assessed by IHC tissue staining and the ELISA method in patients’ blood samples [41,42].

The iTME of GC was examined in the context of B7H4 expression. Similarly, as observed in ESCC, B7H4 expression positively correlated with Foxp3 Tregs in gastric cancer tissues [45]. High B7H4 expression on tumour-infiltrating immune cells, but not on tumour cells, was also significantly associated with a lower density of CD8-positive cells and a higher density of TAMs [40]. Shan Z-G and colleagues, investigating the B7H4 and neutrophils in a gastric cancer environment, discovered that the neutrophils are another source of B7H4. B7H4 expression elevates from peritumoural and nontumour tissue neutrophils to significantly higher levels on intratumoural neutrophils [44]. Moreover, when monocytes were circulating in GC patients’ blood, the positive expression of B7H4 after complete resection of the tumour was significantly reduced [43]. It is also indicated that neoadjuvant chemotherapy (NACT) of GC patients induces the presence of high CD4 and CD8 TIL levels and reduces the expression of B7H4 [46].

Further analysis on GC cell lines revealed that for B7H4 overexpression on monocytes, direct contacts between cancer cells and monocytes, but not soluble factors, are required [43]. Downregulation of B7H4 by siRNA suppressed the proliferation of the MGC-803 human gastric cancer cells through cell cycle arrest in the G1 phase and motility. Decreased expression of B7H4 also leads to activation of caspase-3 and caspase-9 and alternating the Bax/Bcl-2 ratio in favour of apoptosis [47]. Detailed studies of neutrophils in a GC environment revealed that GM-CSF activates neutrophils and induces B7H4 expression in neutrophils. That effect occurs through the activation of the JAK-STAT3 signalling pathway by GM-CSF in the GC environment [44].

#### 3.2.3. Pancreatic Cancer

In pancreatic cancer tissues (PDAC), B7H4 is also overexpressed compared with adjacent tissue. The B7H4-positive cancer tissue sample rate varied among studies from 22.1% to 76% cases [48,49,50,51,52]. However, one study reported no difference in the B7H4 expression between cancer and healthy tissue [53]. B7H4 was co-expressed the most frequently with B7H3. Its expression rate on PDAC tumour cells varied from 60.8% to 88%, in contrast to PD-L1, which was positive on approximately 22.8% of tumour cells [49,51]. B7H4 expression is positively associated with poorly differentiated tumours, localisation in the pancreas, and body and tail and lymph node metastases [49]. Nonetheless, several other studies investigating the relation of B7H4 with clinical cancer features did not report similar findings [48,54,55]. There are also some contradictions regarding the prognostic role of B7H4 in PDAC. Chen X and colleagues noted that high expression of B7H4 was related to shorter progression-free survival (PFS) and DFS [51]. In line with that finding, another group reported the impact of B7H4 on worse OS [48]. On the contrary, Loch F and colleagues and Zhu Y et al. showed no association between B7H4 and OS parameters [52,53].

The results of investigating the iTME in relation to B7H4 are contradictory in PDAC. Several studies reported that, similar to other solid cancers, there is a negative association of B7H4 with the rate of CD8 T cell infiltration [51,54,55]. On the other hand, Zhu Y and colleagues did not find such a correlation [53]. Further, they found a significant negative correlation between the infiltration intensity of TAMs and B7H4 expression in tumour cells and no significant correlations between B7H4 expression in TAMs and the infiltration intensity of CD8 T cells [53].

Immune cell-related extracellular traps (ETs) are a form of cell death. They are characterised by the production of extracellular webs of nuclear DNA and granular and cytoplasmic proteins by immune cells after infection, surgery, radiation, or chemotherapy. Neutrophils are known to produce ETs [56,57]. Neutrophil ETs also mediate the suppression of antitumour immune cells [58]. The study conducted by Chen X et al. aimed to investigate the role of ICs in PDAC concerning ET formation [59]. They found that B7H4 expression was positively associated with neutrophil ET formation, thus contributing to worse PDAC patients’ prognosis [59].

The L3.6p1 PDAC cell line analysis revealed several tumour-associated processes in which B7H4 was involved. Inhibition of B7H4 expression leads to improved cell–cell adhesion and a decrease in pseudopodia formation. Moreover, B7H4 siRNA inhibits cell proliferation, colony formation, and cell migration. Silencing B7H4 also leads to the inactivation of the ERK1/2 mitogenic signalling pathway. It promotes apoptosis by increasing levels of the pro-apoptotic protein Bax, reducing the expression of the anti-apoptotic Bcl-2 protein, and activating caspase-9 and caspase-3 [60].

#### 3.2.4. Cholangiocarcinoma and Gallbladder Cancer

The expression of B7H4 protein has been detected in 49.1% of cholangiocarcinoma (CCA) tissues, while 21.1% of chronic inflammatory bile duct tissue samples and biliary adenoma samples stained negative for B7H4. B7H4 is predominantly expressed in the infiltrating mononuclear cells rather than the epithelial cells of the bile duct and the cell membrane of tumour cells [61,62]. Interestingly, the sB7H4 concentration in the bile of early-stage CCA is significantly higher than that in benign biliary strictures. Compared with conventional serum tumour markers CA19-9, CA12-5, and CEA, the diagnostic and differential diagnosis performance of bile sB7H4 at the cut-off levels were significantly higher in differentiating benign changes from malignant ones than the first three [63]. B7H4 was significantly associated with vascular invasion, lymph node metastasis, TNM stage, and poor tumour differentiation [61,62,63]. Further, the high expression of B7H4 was related to shorter OS, DFS, and recurrence of CCA [63].

B7H4 expression in the tumour cells is inversely correlated with the density of CD8 T cells in the tumour stroma. On the contrary, it is neither correlated with the density of CD8 T cells in the tumour nest nor the CD4 T cells in the tumour stroma or nest [61].

After silencing the expression of B7H4 in QBC939 and RBE ICC cell lines, the cells have been co-cultured with CD8 cytotoxic T cells. As a result, the cytotoxicity of CD8 T cells has been markedly improved by the knockdown of B7H4 in QBC939 and RBE cells [61]. Another group conducted further analysis of these two cell lines. The data revealed that high expression of B7H4 promoted the proliferation, invasion, and migration of ICC cells. B7H4 could significantly promote tumour growth and tumour progression of ICC cells in vivo. Tumour samples expressing high B7H4 tended to have an upregulation of Vimentin and Snail, and the downregulation of E-cadherin and downregulation of B7H4 in ICC cells had the opposite effect. Lastly, high levels of B7H4 contribute to the inhibition of apoptosis and activation of the ERK1/2 signalling pathway [62].

The number of studies tackling gallbladder cancer (GBC) in relation to B7H4 expression is minimal. The positive rate of B7H4 varied from 57% to 69.0% of GBC samples, and there was no expression in chronic cholecystitis samples. B7H4 was co-expressed with B7H3 (67–71% of GBC cases) [64,65]. B7H4 expression rate had a negative correlation with clinical stages of gallbladder carcinoma, and its expression decreased with the increase in clinical stages [65]. On the contrary, another study reported that the B7H4 expression was associated with the TNM stage [64]. Results assessing the impact on OS parameters between the B7H4 high-expression group and the B7H4 low-expression group are limited and inconsistent [64,65]. High B7H4 was associated with a lower density of CD8 TILs in GBC. However, there is no difference regarding Treg density [64].

#### 3.2.5. Hepatocellular Carcinoma

The available data regarding B7H4 expression in hepatocellular cancer (HCC) are highly varied. Its expression rate in HCC was reported to be 1% to 73% in HCC-evaluated cases using the IHC method [66,67]. Besides its overexpression in cancer tissue samples, the sB7H4 form was also reported to be significantly elevated in the sera of HCC patients compared with healthy volunteers. The expression of B7H4 in HCC tissue samples and soluble form from sera of HCC patients was positively associated with several clinicopathological features [68,69]. The vascular invasion, lymph node metastasis, poorly differentiated tumours, and TNM stage were related to B7H4 overexpression [66,68,69,70]. Further, with the stage progression, the levels of sB7H4 increased; inversely, after transcatheter arterial chemoembolisation, its levels substantially decreased [71]. In prognostic analysis, the B7H4 overexpression was related to shorter OS and higher cancer recurrence probability [66,68].

Detailed studies on HCC cancer cell lines align with findings from other gastrointestinal solid cancers. The results of B7H4 knockdown in SMMC7721 and HepG2 HCC cell lines proved its involvement in cancer migration, invasion, stemness of cancer cells and impairing CD8 T cell-mediated cytotoxicity [66]. The downregulation of B7H4 also leads to the increasing number of apoptotic cells by increasing the levels of Caspase-3, Caspase-7, Caspase-8, PARP, and Bax protein and decreasing the levels of survivin and Bcl-2 [66,72]. Moreover, B7H4 downregulation decreased the proliferation of cancer cells by arresting the cell cycle in the G0/G1 phase. Hao TT and colleagues also indicated that inhibiting B7H4 expression promotes autophagy through the PI3K signalling pathway [73]. Further, analysis of B7H4 knockdown in mouse models confirmed its role in cancer progression, where the B7H4 knockdown mice had significantly lower tumour cell density, smaller tumours, and higher areas of necrosis in tumours compared with wild-type mice [72].

#### 3.2.6. Colorectal Cancer

Similarly, like in other gastrointestinal tumours, in colorectal cancer (CRC) tissue samples, B7H4 was also overexpressed in contrast to adjacent healthy tissue. The rate of B7H4-positive cancer samples varied from 29.1% up to 80% [6,74,75,76]. The rate of triple positive CRC cases for B7H4, B7H3, and PD-L1 staining was 13.6% and, for B7H4 and B7H3, it was 6.3%, and that of double positive CRC cases with PD-L1 and B7H4 was 4.6% [75]. The overexpression of B7H4 in CRC was correlated with several clinicopathological features, such as lymph node metastasis, metastasis to the liver, poorly differentiated tumours, and TNM stage [74,75,76,77,78,79]. Our previous study indicated significantly more frequent expression of B7H4 in microsatellite stable tumours (MSS). The MSS status marks usually “cold” iTME. On the contrary, the microsatellite instable tumours (MSI) are the markers of the effectiveness of anti-PD-1 therapy in immunological “hot” tumours [6].

A few articles tackle the issue of the prognostic role of B7H4 in CRC. However, the results are contradictory. Several studies indicated that high B7H4 expression in serum and cancer tissue samples is related to worse OS parameters, shorter DFS, and higher cumulative recurrence rates [76,77,78,80]. On the other hand, Lu Z and colleagues obtained results where B7H4 had no impact on OS and DFS parameters [75]. However, our recent meta-analysis seems to support the thesis of B7H4 correlation with worse prognosis [81].

In our previous study, the expression of B7H4 was negatively correlated with tumour-infiltrating lymphocytes but not with CD8 T cells, and the latter finding was confirmed by other groups [6,75,80]. On the contrary, Peuker K and colleagues observed the relation between B7H4 high expression and lower CD8 T cell infiltration [82]. B7H4 positive expression was correlated with high CD3 T-cell density (CD3 is present on both CD4 and CD8 T cells) and TAMs [75,79]. In our study, we investigated the cytokinome of CRC, and in B7H4 positive tumour tissue homogenates, we observed a negative correlation between B7H4 and several antitumour cytokines and chemokines: IL-9, IL-18, CXCL10, and CXCL9 [6].

B7H4 knockdown in HT29, HCT 116, SW620, and LOVO CRC cell lines leads to a decreasing proliferation rate of tumour cells, promoting apoptosis and the inhibition of migration through decreasing expression of MMP-2 and MMP-9 [78,80,83]. On the contrary, B7H4 overexpression might mediate EMT through the WNT signalling pathway and promote the expression of stemness-related proteins [78]. Further research indicated that the expression of B7H4 may be regulated by the PI3K/AKT/mTOR pathway [83]. Moreover, the immune checkpoint expression might also be regulated by local microbial signals within the tumour environment and be further integrated by myeloid cells. Myeloid calcineurin promotes NFAT-dependent IL-6 transcription, which then acts on tumour cells and supports epithelial B7H3 and B7H4 expression in a STAT3-dependent manner [82]. Another study established that the IFN-γ/IRF1/B7H4 regulatory axis regulates the expression of B7H4 and induces an immunosuppressive effect by promoting the release of GzmB from CD8 T cells and promoting apoptosis in CD8 T cells [77].

### 3.3. Urinary

#### 3.3.1. Renal Cell Carcinoma

It has been reported that high expression of B7H4 is a poor prognostic factor in renal cell carcinoma (RCC) [84]. B7H4 was expressed in tumour and endothelial cells, suggesting its potential role in tumour progression and neovascularisation [85]. The average rate of RCC tumours expressing B7H4 was 60% and did not differ significantly between studies [84,86]. Fukuda T. et al. indicated that increased preoperative serum concentrations of B7H4 in patients with RCC are associated with advanced stage in TNM scale, lymph nodes involvement, high grade, vessel invasion, tumour infiltration by lymphocytes, and decreased survival [84]. According to a study conducted by Azuma T., elevated B7H4 levels in serum are positively related to a number of peripheral neutrophils [87]. The co-expression of B7H4 with other members of the B7 family seems to be crucial for RCC prognosis—it was demonstrated that patients with tumours expressing both B7H4 and B7H1 had decreased survival in comparison to those with no B7H4/B7H1 expression or positive staining only for B7H4 or B7H1 alone. Additionally, a high level of B7H4 correlates with an increased risk of disease progression after nephrectomy [88].

The last studies noticed that the pattern of B7H4 expression in RCC could be modulated by tyrosine kinase inhibitors (TKIs) and mTOR inhibitors combined with immunotherapy to improve the response rate in patients. Emaldi M. et al. [89] showed that in RCC lines, B7H4 expression increases after treatment with TKI and mTOR inhibitors. In contrast, the simultaneous inhibiting of the B7H4 gene and applying TKI/mTOR inhibitors resulted in increased inhibition of RCC cell growth compared with using TKI/mTOR inhibitors alone [90]. These findings may suggest the involvement of B7H4 in responsiveness to therapies targeted tyrosine and mTOR kinases such as sunitinib, sorafenib, pazopanib, aksytynib, temsirolimus, and everolimus.

Additionally, based on mRNA-sequencing data from TCGA, B7H4 may create a typical expression pattern with chemokines and metalloproteinases, including CXCL1/2/3, CXCL8, CCL20, and MMP7. Consistent with these in silico findings, Li A. demonstrated that blocking CXCL8 in the RCC mouse model leads to tumour growth inhibition, suggesting that CXCL8 mediates the process. In this study, B7H4 was also shown to recruit tumour-infiltrating neutrophils through CXCL8 [89].

#### 3.3.2. Urothelial Cancer

In urothelial cancer, B7H4 was found on the surface of cancer cells, CD68 macrophages, while its expression was limited in TILs and normal urothelial cells [91]. In a study by Liu WH, B7H4 expression was found in 49% of examined tumours; its concentration in serum was also significantly increased compared with the healthy control group [92]. B7H4 was reported to be overexpressed in bladder cancer and increase the capability of cancer cells to migrate and invade [93], while B7H4 blocking in the bladder cancer cell line resulted in increased cytotoxic activity of T cells [92]. The upregulation of B7H4 expression was found to promote EMT, as confirmed by the B7H4-mediated downregulation of E-cadherin expression and upregulation of Vimentin. Also, the expression of well-known EMT inducers Twist1 and Snail was revealed to be increased by B7H4 upregulation and accordingly decreased by B7H4 knockdown [93]. Liu Z et al. identified a correlation between upregulated expression of B7H4, tumour mutation burden (TMB) and higher responsiveness to anti-PD-L1 therapy in bladder cancer patients. Although high expression of B7H4 is associated with an unfavourable prognosis, this subset of patients benefits more from immunotherapy [5]. Blocking of B7H4 using a monoclonal antibody in a mouse model of muscle-invasive bladder cancer (N-butyl-N-(4-hydroxybutyl)-nitrosamine (BBN)) leads to reduced tumour size, an increase in number of CD8 T cell infiltrating the tumour, and a decrease in Tregs infiltration. Blocking both PD-L1 and B7H4 by monoclonal antibodies resulted in less advanced tumour stage, tumour growth inhibition, and enhanced necrosis within the tumour, highlighting the potential usability of B7H4 inhibitors in bladder cancers unresponsive to the PD-1/PD-L1 blockade [94].

#### 3.3.3. Prostate Cancer

In prostate cancer (PCa), increased expression of B7H4 was reported to be positively correlated with advanced clinical stage, T feature, increased risk of disease recurrence, and decreased OS rate, and it was an independent negative prognostic factor. IHC staining showed that B7H4 is expressed in the cytoplasm and membranous of prostate cancer cells. Analysis of the co-expression of B7H4 with other genes revealed that in PCa, B7H4 expression is related to genes regulating cancer cells’ stemness and influences the PI3K/Akt signalling as well as other pathways associated with cell cycle control [95]. Cancer stem cells (CSCs) are characteristic of advanced PCa. It refers to a group of tumour cells that can self-renew and have increased potential to invade and metastasise, resulting in resistance to systemic therapy [96]. Elevated expression of B7H4 and other immune checkpoints involving PD-L1, IDO-1, and OX40L was found in the blastic type of PCa bone metastases, suggesting B7H4 involvement in PCa bone metastases [97].

### 3.4. Gynecological Cancers

#### 3.4.1. Cervical Cancer

B7H4 does not show expression in healthy cervical epithelium, but the protein has been detected in cervical cancer [98,99,100]. In a study by Huang et al., B7H4 was expressed mainly in the cytoplasm of cancer cells, and 80.56% of specimens exhibited B7H4 expression [101]. Wang and colleagues also found mononuclear cells of the tumour microenvironment to be positive for B7H4 [100]. On the other hand, Zong et al. detected B7H4 expression in tumour cells in 44.8% of cervical cancer samples but not in immune cells [102]. B7H4 protein levels may rise along with inflammation intensity, as lower expression rates were detected in CIN II patients, higher in CIN III, and the highest in cervical cancer patients [103].

Measuring B7H4 levels in serum (sB7H4) has also brought noteworthy results. sB7H4 levels were positively correlated with B7H4 expression in vascular endothelial cells, mesenchymal fibroblasts, and cancer cells of the HPV-positive inflammation group, CIN, and cancer patients, meaning that sB7H4 likely originates from cervical lesions [104]. sB7H4 concentrations increased similarly to B7H4 expression rates in the cervix, showing the highest values for patients with cervical cancer [99]. The protein concentration was also elevated in patients suffering from HPV-positive inflammation cervical disease compared with HPV-negative cases [104]. Notably, measuring sB7H4 levels in the blood could determine CIN or cervical cancer occurrence with a sensitivity of 93.33 and a specificity of 87.50%, and similar results were obtained by Qiu et al. These data suggest that sB7H4 could serve as a reliable early marker of cervical cancer [99,104].

There is a positive association between the expression of B7H4 and B7H3 [98,101]. Moreover, Zong et al. found that B7H4 in tumour cells correlated positively with VISTA expression in immune cells, with 43.1% double-positive cases [102]. According to Chen and colleagues, B7H4 is associated negatively with PD-L1, EGFR, and the expression of most immune markers [105]. Drug databases reveal that high B7H4 expression is an indicator of worse responses to anti-ERBB, antiangiogenic, and immunotherapy. However, at the same time, such tumours are more sensitive to traditional antitumour drugs. It might indicate that B7H4 is a marker of limited treatment options in cervical cancer [105].

Several authors reported no connection between B7H4 expression in cervical cancer and most clinicopathological features of the patients [98,99]. However, certain studies mention correlations between the protein levels with the FIGO stage and tumour size [101,102]. According to Zong and colleagues, B7H4 expression is correlated with small tumour sizes and more prominent lymphovascular space invasion [102]. Chen et al. found high B7H4 concentrations associated with worse OS and RFS [105]. On the contrary, Zong et al. reported B7H4 expression as associated with better RFS [102]. Moreover, B7H4 and VISTA double-positive tumours had significantly better relapse-free survival (RFS) and disease-specific survival (DSS). The double-negative B7H4 (in tumour cells) and VISTA (in immune cells) served as the sole prognostic factor for poor RFS and DSS in PD-L1-negative patients [102]. Such results indicate that B7H4 has a prognostic potential in cervical cancer. However, its role in the disease may be more complex than expected—except for its immunoinhibitory function; it may also inhibit tumour growth, contributing to better clinical outcomes, which should be considered and further explored in future reports [106].

In B7H4-positive specimens, the average number of CD8 T infiltrating cells was significantly decreased compared with the B7H4-negative cases, and higher B7H4 expression corresponded to immuno-cold tumours. There was also an increase in Tregs in B7H4-positive cases. Additionally, the CD4 T/CD8 T ratio and the relative number of CD25+Foxp3+T cells were higher after co-culturing with B7H4. Moreover, B7H4 upregulation corresponded to decreased IFN-γ and IL-2 secretion but also increased IL-10 and TGF-β1 concentrations. These results imply that B7H4 may silence immunological responses in cervical cancer [100,101,107]. sB7H4 in serum correlated with an elevated number of Tregs in the peripheral blood of cervical cancer patients, suggesting its potential for determining the immunological status of patients [104].

Wang and colleagues demonstrated that co-culturing with recombinant B7H4 significantly reduces the number of T-cells in the S phase of the cell cycle and Ki67-positive cells compared with controls [100]. This indicates that B7H4 could impair T-cell proliferation, thus inhibiting antitumour responses [100]. Moreover, B7H4 upregulation increased cell viability and augmented S to G2/M phase transition, while *VTCN1* silencing led to cell arrest in the G0/G1 phase and reduced cell viability. B7H4 knockdown also downregulates cell cycle regulators—pRB, E2F, P16, and P21. Compared with the controls, *VTCN1* silencing increased early and late apoptosis and upregulated the levels of apoptosis-related proteins. Moreover, *VTCN1* targeting could reduce cell migration and invasion. In the cells with silenced B7H4, the mRNA levels of MMP-2, MMP-9, and VEGF—molecules related to cancer cell migration and invasion—were reduced [99]. On the other hand, co-culturing with B7H4 overexpressing cell lines decreased apoptosis in monocytic U-937 cells [107]. These results indicate that targeting VTCN1 could serve as a therapeutic strategy in cervical cancer, but its previously mentioned antitumour activity should also be taken into account. Notably, B7H4 silencing increased tumour suppressor Rb mRNA and downregulated E7 mRNA—E7 is an HPV oncoprotein inhibiting Rb function [99,108]. The opposite effects were observed upon VTCN1 upregulation, suggesting that B7H4 may take part in the E7/Rb pathway during HPV infection, contributing to cancer development. The role of B7H4 in mediating HPV-induced oncogenesis is also a promising area for future studies, as targeting the molecule may have clinical potential for preventing cervical cancer in such cases [99].

#### 3.4.2. Ovarian Cancer

B7H4 can be found in most ovarian cancer specimens, while no B7H4 expression has been observed in noncancerous ovarian tissues [109,110,111,112]. In a study conducted by Hwang et al., 94% of samples obtained from patients suffering from ovarian serous carcinoma were positive for B7H4 [111]. Moreover, Zheng and colleagues uncovered that the expression rate of B7H4 in benign ovarian cancer tissues was lower (20%) than in ovarian cancer in general (80%) [109]. IHC of ovarian cancer samples revealed B7H4 staining in the cytoplasm and membrane of cancer cells. However, its expression in stromal cells was weak or not detectable, meaning that B7H4 expression in ovarian cancer is mainly limited to tumours. B7H4 expression on APCs was also deficient. According to the TCGA ovarian serous cystadenocarcinoma dataset, the most robust B7H4 expression occurs in ovarian cancer’s immunoreactive/C2 and differentiated/C4 subsets, characterised by high T-cell infiltrations [113,114,115]. Although Mach et al. detected sB7H4 in the serum of 12 out of 85 (14.1%) epithelial ovarian cancer (EOC) patients [116], Lan and colleagues demonstrated in their meta-analysis that serum B7H4 has sufficient for clinical use specificity and sensitivity in detecting ovarian cancer [117].

There was a significant positive correlation between the expression of B7H4 and other proteins, such as CD24, PCNA, and IDO-1 [109,114]. CD24 is a protein associated with tumour growth and the migration of cancer cells, and its overexpression corresponds to a poorer prognosis in ovarian cancer [118]. PCNA participates in DNA replication and is a cell proliferation marker [119]. IDO1 is another protein silencing immune responses in cancer [120]. Niu and colleagues found that 49.1% of all high-grade serous ovarian carcinoma (HGSC) specimens investigated exhibited the expression of both IDO-1 and B7H4 [114]. There was also a positive correlation between B7H4 and Tim3—a regulatory molecule playing an immunoinhibitory role in cancer [121]. The interplay between B7H4, IDO1, and Tim3 has not been studied in detail. Therefore, it would be interesting to shed more light on the role of these proteins in the modulation of the immunological landscape in OC [114]. There was no correlation between B7H4 and PD-L1 expression; however, PD-L1 was more prominent in APCs from tumours with high B7H4 expression, suggesting alleviated immunological responses in TME in the event of higher B7H4 expression [113]. Notably, B7H3 and PD-L1 exhibited different expression patterns than B7H4, as they were detected mainly in the stromal compartment of ovarian cancer. MacGregor and colleagues suggest B7H4 as an attractive target for OC therapy, especially combined with the simultaneous targeting of molecules in stromal cells (for instance, PD-L1) [110].

The expression rate of B7H4 was increased in poorly or moderately differentiated cancer tissues [109]. B7H4 levels were also associated with a more advanced TNM stage and significantly poorer OS than the B7H4-negative group [111,114]. Moreover, patients with the expression of B7H4 in serum displayed poorer OS, and sB7H4-positivity was an independent prognostic factor for OS in ovarian cancer. This means that B7H4 could be related to a more aggressive course of ovarian cancer, and detecting sB7H4 in OC patients could have a prognostic value for the disease [116]. Additionally, elevated B7H4 expression was observed in drug-resistant patients, and sB7H4-positive patients displayed more common platinum resistance than the sB7H4-negative group [114,116].

Several studies showed no association between B7H4 expression and T-cell or B-cell infiltrations in TME [113,114]. However, Hwang et al. reported that B7H4-positive IHC staining was associated with an increased density of stromal immune infiltrations compared with the B7H4-negative group [111]. In another study, higher B7H4 expression corresponded to more prominent CD11c+HLA-DRhigh APCs in the tumour microenvironment, and the authors suggested that B7H4 affects APC recruitment but does not influence other lymphocytes [113].

The role of VTCN1 in OC needs to be clarified. The TCGA dataset revealed that in serous ovarian adenocarcinomas, B7H4 mRNA levels correlated positively with IL6, IL-10, TGFB1, and IFNG mRNA expression, suggesting associations with proinflammatory and immunoinhibitory mechanisms. There was also a positive association between B7H4 mRNA and the expression of various chemokines (CCL2, CCL4, CCL5, CCL8, CXCL17, CXCL10, CXCL11, and CXCL17), as well as ACKR2 mRNA [113]. ACKR2 can bind with CC-type cytokines, contributing to their degradation, meaning that their function may be altered in B7H4-high ovarian cancer [122]. The mentioned cytokines did not increase VTCN1 expression in vitro, indicating that other factors may be necessary for B7H4 induction in ovarian cancer. Although CXCL17 is an angiogenic chemokine that induces VEGF expression, no correlations between B7H4 and VEGF levels were reported in the study [113,123]. On the other hand, B7H4 expression correlated positively with mRNA levels of CSC-related biomarkers, CD24, CD44s, and CD133. Importantly, B7H4-targeting antibodies show promising antitumour effects in ovarian cancer models, and clarifying VTCN1 function can be crucial for obtaining optimal treatment effects [112,124].

#### 3.4.3. Endometrial Cancer

B7H4 is also upregulated in endometrial cancer (EC) [125,126]. Zong et al. demonstrated B7H4 expression in 71.5% of EC patients [126], while in other studies, nearly all EC samples were B7H4-positive [112,127,128]. B7H4 showed cytoplasmatic and membranous expression and appeared solely in cancer cells [126]. Gorzelnik and colleagues found that in 46% of patients, more than 60% of tumour cells expressed B7H4 [127]. Moreover, the expression rate of B7H4 turned out to be higher in nonspecific molecular profile (NSMP) and p53mut than in other molecular subtypes of endometrial cancer [126].

The expression rate of B7H4 in tumour cells was negatively associated with B7H3 expression in immune cells but not in cancer cells [126]. Bregar et al. found a significant co-expression between B7H4 and PD-L1 in high-grade EC patients, and this co-expression pattern occurred mainly in high-grade endometroid carcinoma and carcinosarcoma. Such results suggest that combined therapy targeting B7H4 and PD-L1 could benefit high-grade endometrial carcinomas [129]. On the contrary, no correlation of B7H4 with PD-L1 levels was observed in another study [126].

Interestingly, in another study, B7H4-positivity correlated with age, being more prominent in 58-year-old patients and older. Additionally, B7H4 detection corresponded to more favourable clinicopathological characteristics, including early stage, low grade, absence of lymphovascular space invasion, and diminished myometrial invasion [126]. However, these results were not confirmed in other studies [127,129]. There was also a negative correlation between *VTCN1* expression and tumour mutation burden (TMB) but no association between B7H4 and mutation prevalence or microsatellite instability [128,129].

Zong et al. showed that B7H4-positivity was associated with better RFS and DSS; it was also an independent prognostic factor for longer DSS in EC [126]. In contrast, Gorzelnik and colleagues showed that high B7H4 expression corresponded to a worse OS than B7H4-low patients [127]. *VTCN1* hypomethylation and upregulation were associated with a less favourable prognosis [130]. Moreover, the five-year survival rate was poorer in the case of high B7H4 expression than in the B7H4-low group (69% compared with 92%, respectively) [127].

Several authors found no correlation between B7H4 expression and T-cell infiltrations in EC [126,129]. On the other hand, hypomethylation and high B7H4 expression were positively associated with resting memory CD4 T cells and negatively with CD8 cells or activated memory CD4 T cells [130]. More studies are needed to elucidate the influence of B7H4 on the immunological environment in endometrial cancer. Its molecular role in the disease is also not known. More reports concerning B7H4 function in EC are needed to establish its utility in the context of endometrial cancer treatment and its prognostic potential.

### 3.5. Head and Neck Cancers

Recent reports show contradictory results regarding the expression of B7H4 in head and neck squamous cell carcinoma (HNSCC). In oral squamous cell carcinoma (OSCC) cell lines, B7H4 mRNA and protein expression were more than twofold higher than in human oral keratinocytes [131]. On the other hand, Borgmann et al. found that among 395 specimens with interpretable B7H4 staining, most cases were B7H4-negative [132]. B7H4-positive samples displayed the molecule expression in the cell plasma membrane and cytoplasm. Additionally, the authors found moderately higher B7H4 expression in normal tissues than in tumours [132]. In salivary gland carcinomas (SGCs), B7H4 expression was detected in 50% of tumours, with 14% of samples showing high B7H4 levels. The highest expression rate (94%) was observed for adenoid cystic carcinoma (ACC), while all acinic cell carcinoma (AciCC) specimens were B7H4-negative. B7H4 expression did not occur in normal salivary glands [133].

B7H4 expression was associated with other proteins that can be prognostic markers in HNSCC. A significant positive correlation was reported between the levels of B7H4 and CD168 and DNA methyltransferase-1 (DNMT1)—molecules related to worse survival in OSCC [134,135]. Although the expression of B7H4 is associated with proteins that contribute to worse outcomes in OSCC, its prognostic value in head and neck carcinomas is unclear. In a study by Borgmann and colleagues, B7H4 expression was not correlated with OS in HNSCC. However, in oropharyngeal SCC (OPSCC) samples positive for the HPV surrogate marker p16 (p16+), there was a trend towards worse survival in B7H4-positive patients, but it failed to reach statistical significance [132]. In ACC, B7H4 expression correlated with the solid subtype and appeared in high levels in 94% of such samples. High B7H4 levels in ACC were related to poorer survival prognosis and worse median OS than B7H4-low or -negative tumours. This tendency was not observed for other SGCs [133]. Such results imply that B7H4 has a prognostic value for ACC.

In OSCC cell lines, B7H4 silencing led to a decrease in cell migration, invasion, and proliferation. Moreover, when *VTCN1* was silenced, the expression of M1 macrophage markers increased significantly, and the opposite tendency was observed for M2 polarisation markers. Targeting B7H4 also resulted in a decreased expression of PD-1 and reduced STAT3 phosphorylation. On the other hand, the induction of the PD-1/STAT3 pathway reversed the results of B7H4 knockdown both in vitro and in vivo. This observation implies that B7H4 likely modulates macrophage polarisation and contributes to the anti-inflammatory microenvironment in OSCC via the PD-1/STAT3 signalling. Targeting B7H4 could also decrease xenograft tumour weight and volume in vivo, but again, these effects were reversed upon PD-1/STAT3 induction using Colivelin. B7H4 promotes a more aggressive phenotype in cancer cells and silences antitumour responses in OSCC via PD-L/STAT3 signalling, thus making it an attractive target for OSCC therapy [131].

### 3.6. Lung Cancer

B7H4 was elevated in the serum of patients with non-small cell lung cancer (NSCLC) and co-expressed with another immune checkpoint, Siglec-15, in tumour tissue [136,137]. Available data regarding B7H4 expression and its role in small-cell lung cancer (SCLC) are minimal. The rate of B7H4-positive SCLC tumour cells varied from 2.6% to 5.83% [138,139]. However, another group reported a 74.77% rate of B7H4-positive tumours [140]. High B7H4 levels correlated positively with metastases and shorter OS [139,140]. In the adenocarcinoma subtype of NSCLC, positive staining for B7H4 was detected in 44.9% of tumours, mainly in the cytoplasm and membrane of cancer cells. What is worth noting is that B7H4 was rarely co-expressed with other immune checkpoints. High B7H4 expression was more prominent in tumours with EGFR mutation [141,142,143]. In squamous cell carcinoma, B7H4 is co-expressed with B7H3. A positive association between increased B7H4 expression and poor survival was also confirmed in SCC [142].

In the cell line model of lung cancer (the IGF1/IGF1R) axis was shown to induce B7H4 expression through the MEK/ERK1/2 pathway. A mouse model subsequently confirmed this finding by demonstrating enhanced tumour growth and decreased infiltration of T cells within the tumour with IGF1 overexpression [144]. In murine models, high B7H4 expression was related to metastases presence and decreased amount of CD8 T cells. B7H4 was demonstrated to induce PD-L1 expression—its inhibition in cancer cells expressing both B7H4 and PD-L1 reduced T cell apoptosis [137].

### 3.7. Central Nervous System Malignancies

Chen et al. found that 54.1% of glioma tissue samples were B7H4-positive, and 19.6% displayed high molecule expression, as revealed by IHC [145]. The expression rate of B7H4 in glioblastoma multiforme (GBM) was 53.8%, while in lower-grade gliomas (LGG), 54.2% of samples were positive for B7H4. In these diseases, intense B7H4 staining was observed in 14.1% and 24.4% of specimens, respectively [145].

The expression of B7H4 correlated negatively with the expression of numerous immune checkpoint genes, including IDO1, CTLA4, PD-1, and TIM-3. Additionally, there was a negative association in TCGA and CGGA cohorts between PD-L1 and B7H4 expression. Moreover, according to Chen and colleagues, only 2% of glioma patients exhibited high expression levels of both B7H4 and PD-L1. On the other hand, 61% of tumours showed double-low expression of the two molecules, 20% of samples exhibited PD-L1-low and B7H4-high expression, and 17% of specimens were classified as PD-L1-high and B7H4-low. The authors suggested a novel molecular classification of gliomas based on three mentioned subtypes (double-low, PD-L1-low, and PD-L1-high). There was a correlation between the mentioned subtypes and the histological type of the tumour. Such a classification could help determine patients’ responses to potential therapies targeting B7H4 [145].

Interestingly, high B7H4 expression was more common in male than female patients and correlated with WHO grade (high B7H4 levels were most common in WHO III tumours) and with the histological type (most common in astrocytoma) [145]. In GBM, high B7H4 expression corresponded to worse OS and (PFS) in patients after irradiation, meaning it could be considered a prognostic marker for GBM [146].

High PD-L1 expression was associated with significantly more abundant immune infiltrations in tumours than B7H4-high patients. There was also a negative association between high B7H4 levels and the number of TILs and TAMs in the tumour microenvironment [145].

Significantly, B7H4 can also influence the results of other antitumour therapies. Yen and colleagues found that low B7H4 expression in glioma corresponded to increased sensitivity to dendritic cell-based vaccination (DCV) and correlated with better OS after DCV treatment [147]. Moreover, B7H4 plays a role in patients’ response to radiotherapy. Irradiation increased exosomal and membranous levels of B7H4 in GBM cells compared with controls. Tian et al. revealed that irradiation leads to STAT3 phosphorylation via ATM and likely enhances STAT3 binding to the *VTCN1* promoter region, causing its upregulation. Increased B7H4 from irradiated GBM exosomes modulates Th1 cell differentiation and takes part in the induction of anti-inflammatory responses. Additionally, in differentiating Th1 cells, B7H4 inactivated STAT1 pathway contributing to augmented differentiation towards regulatory T-cells. The irradiation-induced decrease in Th1 cells and simultaneous increase in Tregs and PD-1+ TIM-3+ exhausted CD8 T cells could also be reversed upon B7H4 knockdown. Moreover, B7H4 silencing in mice reduced tumour growth after irradiation and increased their survival, but the effect did not occur in immune-deficient mice. Together, these results indicate that B7H4 decreases GBM cells’ radiosensitivity via exosomes and promotes anti-inflammatory responses [146].

To summarise the most relevant data regarding B7H4 expression rate, prognosis, and potential role of B7H4 in tumours, we provided Table 1. Further, we collected the evidence of B7H4 expression influence on the immunological landscape in tumours in Table 2.

## 4. B7H4 Targeting Immunotherapies

**Figure 3:**
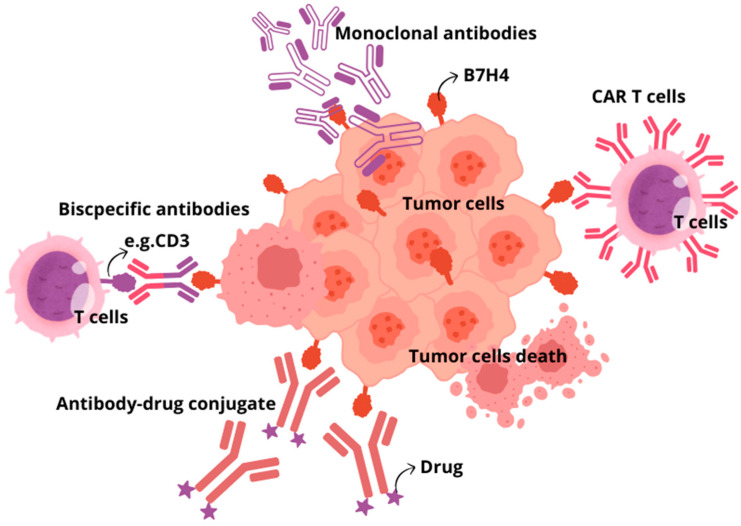
Basic overview of immunotherapeutic approaches targeting B7H4. Monoclonal antibodies; Adoptive cell therapy, CAR T cells; Antibody–drug conjugates; Bispecific antibodies.

There are several therapeutic approaches for using B7H4 as a target. Strategies under consideration include monoclonal antibodies, bispecific antibodies, antibody–drug conjugates, and CAR T cells, as well as inhibiting the B7H4 glycosylation (Figure 3) [148]. Monoclonal antibodies targeting immune checkpoints (ICIs), CTLA-4, PD-1, and PD-L1, are still among the most common and effective therapeutic strategies. However, due to the growing use of ICIs, the prevalence of immune-related adverse effects (irAEs) also increases. The most common irAEs include gastrointestinal, endocrine, and dermatological toxicity, while fatal irAEs are neurological, cardiovascular, pulmonary, and renal toxicity. It is worth noting that ICI combination therapy with the use of more than one ICI is related to a higher risk of irAEs than in ICI monotherapy. Despite the impressive successes of ICI application, it is estimated that in therapy of malignant neoplasms, only 30% of patients respond to treatment blocking the PD-1/PD-L1 axis, which is currently most common used immunotherapy strategy. Additionally, the usefulness of ICIs is limited by other factors, including the presence of cancer stem cells or different pathways of immune evasion [149]. Therefore, searching for new immune checkpoint inhibitors is crucial [150]. As B7H4 mediates immune evasion in tumours in a way other than PD-1, PD-L1, and CTLA4, blocking this pathway would restore antitumour immunity. Several preclinical studies proved that blocking B7H4 had significant therapeutic efficacy in various syngeneic murine models. B7H4 blockade in breast cancer murine models resulted in sustained inhibition of tumour growth, and combination treatment with anti-PD-1 antibody exerted a synergistic effect, resulting in maximal inhibition of tumour growth [151]. To the best of our knowledge, there is no clinical trial establishing dual immune checkpoint blockade targeting B7H4 and immune checkpoint other than PD-1/PD-L1. In comparison to ICIs, more encouraging results regarding targeting B7H4 in solid tumours were obtained with bispecific antibodies. BsAbs are molecules that simultaneously bind to two different and distinct antigens, providing a precise immune response to target tumour cells. The first bsAbs used in tumour immunotherapy aimed at activating T lymphocytes towards cancer cells through affecting the interaction between CD3 receptor on T cells and cancer-specific antigens on tumour cells. It results in promoting the interaction of MHC with T-cell receptors, which leads to T lymphocyte activation. In this way, bsAb-targeted proteins present on the surface of tumour cells and bind to selected receptors expressed on immune effector cells. The main limitations of bsAbs application involve short half-life of bsAbs and adverse toxic effects, including cytokine release syndrome and organ toxicities, similarly to ICIs. Trials evaluating bispecific antibodies targeting B7H4 are ongoing. Anti-B7H4/CD3 bsAbs applied in a breast cancer humanised mouse model led to immediate and strong antitumour activity tumours and CD8 and granzyme B+ CTL infiltration into the tumour, and there were no adverse effects after long-term observation [152]. The ABL103—a bispecific antibody that binds to B7H4 and 4-1BB (4-1BB is a highly potent costimulatory molecule expressed in T and NK cells)—simultaneously potently inhibited tumour progression in a dose-dependent manner and showed a higher rate of complete remission. Moreover, mice were free from tumour recurrence three months after the cessation of the ABL103 treatment [153]. Similarly, the application of anti-B7H4/IL-15 bispecific antibodies against cold cancers like triple-negative breast cancers and ovarian cancer turned to improve immunogenicity within TME by enhancing the proliferation of CD8 T cells and boosting immune cell-mediated killing (ADCC) of B7H4+ tumour cells [154]. In antibody-dependent cell-mediated toxicity (ADCC), target tumour cells are opsonised by antibodies that recruit effector cells in the immune system to kill cancer cells in a non-phagocytic-mediated manner. Specified antibodies are capable of binding to the antigens expressed on the surface of tumour cells through a portion of the antigen-binding fragment (Fab) and bind to effector cells through portions of fragment crystallisable region (Fc), thus directly linking target cancer cell and effector immune cell able to destroy tumour cells. Another strategy provides B7H4-directed antibody–drug conjugate (B7H4-ADC). ADCs are new biopharmaceutical agents that use the selectivity of monoclonal antibodies to precisely target and deliver chemotherapeutic drugs to cancer cells, significantly limiting chemotoxicity for healthy noncancerous tissues. Single-dose B7H4-ADC led to tumour regression in 65.5% of breast and ovarian patient-derived xenograft models, with reduced activity in B7H4-low or -negative models. In PARPi and platinum-resistant high-grade serous ovarian carcinoma patient-derived xenograft models, scheduled B7H4-ADC dosing led to sustained tumour regression and increased survival [155]. Another strategy of current immunotherapies that has emerged as a promising and pioneering treatment is chimeric antigen receptor (CAR)-T cell therapy. T lymphocytes with chimeric antigen receptors (CARs) express an engineered receptor able to directly activate T cell signalling domains in an MHC-unrestricted manner. Initially, CAR therapy was successfully applied to treat haematological neoplasm, but currently its significance in targeting solid tumours is under extensive investigation in clinical trials. Nevertheless, this approach exhibits limited effectiveness in solid tumours due to the high heterogeneity of tumour-specified antigens, low penetration of CAR-T cells to target cancer cells in tumour mass, and the presence of target antigens in normal tissue, which contributes to organ toxicity. In line with these findings, B7H4 CAR T cell therapies, despite controlling cancer outgrowth, their long-term engraftment of B7H4 CAR T cells led to mediated lethal, off-tumour toxicity that was likely due to broad expression of B7H4 in healthy mouse organs [156]. Finally, the small-molecule oligosaccharide transferase inhibitor NGI-1, which inhibits the addition of N-glycans to B7H4, can be used as an alternative therapeutic approach to cause its ubiquitylation and degradation. The inhibition of B7H4 glycosylation can be favourably combined with immunogenic chemotherapy and PD-L1 blockade to achieve superior immuno-infiltration of cold tumours and improved tumour growth control [27,157].

Several clinical trials assessing B7H4 targeting immunotherapy using various treatment modalities are ongoing (Table 3). There are three phase I trials establishing the utility of B7H4-ADC-based therapies and one phase I/II trial evaluating anti-B7H4 ADC with anti-PD-1 Ab: Tislelizumab vs. anti-B7H4 ADC alone. SGN-B7H4V, an ADC-type drug, showed a manageable safety profile in patients with advanced solid tumours. Moreover, responses were observed at all tested dose levels and across various tumour types [158]. Two different bsAbs targeting B7H4/4-1BB and B7H4/CD3 are currently being studied in phase I and phase I/II trials, but no results have been posted. One phase I clinical trial assesses FPA150, an anti-B7H4 antibody alone or combined with pembrolizumab, an anti-PD-1 antibody, in patients with advanced solid tumours.

## 5. Conclusions and Future Directions

Major successes with ICI and the potential for substantial clinical effects drive many clinical trials. In the prospect of immunotherapies targeting B7H4, it is essential to establish its co-expression patterns with other immune checkpoints in various types of cancer, as they could affect the effects of such treatment. The B7H4 is frequently co-expressed with the B7H3 immune checkpoint. Therefore, exploring multi-checkpoint blockade in patients who are unresponsive or resistant to PD-1/PD-L1 inhibitors may be helpful. The differential expression patterns of the B7H4 and PD-L1/PD-1 axis are relevant to checkpoint blockade therapy, as identifying which pathway is active in a given tumour may have predictive value for the efficacy of ICI therapy. Moreover, as the overexpression of B7H4 in malignancies usually marks the “cold” tumour immune microenvironment, it opens the therapy option for the vast majority of cancers. “Hot” iTME are observed in a minority of tumours (6–15% of CRC cases), or they are related to specific cancer types (melanoma, RCC) [6,159].

Notably, in conditions such as ESCC, GC, and CRC, where most reports are consistent, B7H4 seems to be a better target for immunotherapy than PD-L1, already used in cancer therapies [6,20,36,37,40,41,76,82]. On the other hand, in malignancies like PDAC, CCA, GBC, and gliomas, the number of reports is minimal and presents some contradictions [51,52,53,61,62,63,64]. However, the evidence suggests that it is vital to continue exploring B7H4 co-expression patterns and surrounding iTME of those malignancies. Particularly, more studies are needed regarding breast and lung cancer as they pose substantial health burdens [30,32,34,139,142,143,160]. For example, the exact role of gynaecological cancer needs further clarification [101,102,103,104,105,106,107,108,109,110,111,112,113,114,115,116,117,118,119,120,121,122,123,124,125,126,127,128,129,130,161]. Some report the beneficial effect of B7H4 on cancer prognosis, but the results are not in line with other studies.

Future studies should focus more on studying the iTME to identify features and critical differences that define distinct classes and subclasses of iTME, emphasising the co-expression patterns of immune checkpoints. Substantial progress in this area requires implementing the highest-resolution methods to assess the total cellular composition (flow cytometry), functional status (single-cell RNA sequencing), and cellular localisation (multidimensional immunohistochemistry) of iTME. This knowledge would help stratify the patients according to tumour type and thus apply cancer-tailored immunotherapies [149].

Moreover, a broader approach to iTME composition and IC expression would probably help to overcome the challenges of resistance mechanisms to ICI. The decreased efficacy of ICI can occur through defects in antigen presentation due to the loss or reduced expression of MHC molecules; the secretion of soluble and exosomal PD-L1 that potentially competes with drug binding; the presence of immunosuppressive cell types in the TME that inhibit T-cell functions; T-cell exhaustion and the expression of alternative inhibitory immune checkpoints; a lack of bacterial diversity or the enrichment of specific “bad” microbes in the gut microbiome [162].

Another developing field in immune oncology is immunometabolism. Reprogramming of the metabolism of the cancer cells drives the metabolic dysregulation of TME, causing partial failure of T-cell-based cancer immunotherapy. Increasing evidence suggests that the metabolic adaptations of T cells determine their function by reprogramming T-cell metabolism through checkpoints like IDO, IL4I1, and SIRT2, resulting in improved anti-cancer immune efficacy [163,164].

We can integrate fundamental immunology insights and clinical observations to develop promising multi-targeting immunotherapies with continued advances in understanding the complexity of iTME, immune checkpoint expression patterns, immunometabolism, and tumour cells’ interplay with those elements.

## Figures and Tables

**Table 1:** B7H4 expression rate, prognosis, and potential role of B7H4 in tumours.

Type of Tumour	Expression Rate	B7H4 Expression and Prognosis	Potential Mechanisms	References
Breast cancer	HR+/HER2+ = 25% HR−/HER2+ = 60%	Unfavourable prognosis in the case of elevated B7H4 expression in tumour tissues	Inhibitory effect on immunological responses	[30,31,34]
Oesophageal cancer	53.8–95.5%	Unfavourable prognosis in the case of elevated B7H4 expression in tumour tissues	Stimulation of cell cycle progression	[20,36,37,38]
Gastric cancer	44.9–80%	Unfavourable prognosis in the case of elevated B7H4 expression in tumour tissues	Inhibitory effect on immunological responses	[40,41,42,45]
Pancreatic cancer	22.1–76%	Unclear	Unclear	[48,49,51,52,55,60]
Cholangiocarcinoma	49.1%	Unfavourable prognosis in the case of elevated B7H4 expression in tumour tissues	Promoting tumour growth and tumour progression, inhibition of apoptosis	[61,62]
Gallbladder cancer	57–69%	Unclear	Lower CD8+ TIL density in GBCs	[64,65]
Hepatocellular cancer	1–73%	Unfavourable prognosis in the case of elevated B7H4 expression in tumour tissues	Inhibitory effect on immunological responses	[66,67,68,69,70,71,72]
Colorectal cancer	29.1–80%	Unclear	Inhibitory effect on immunological responses	[6,74,76,78,80]
Renal cell carcinoma	60%	Unfavourable prognosis in the case of elevated B7H4 expression in tumour tissues	Increase in the number of peripheral neutrophils	[84,86,87]
Bladder cancer	49%	Unfavourable prognosis in the case of elevated B7H4 expression in tumour tissues	Increased ability of cancer cells to migrate and invade and increased cytotoxic activity of T cells	[92,93]
Prostate cancer	Unclear	Unfavourable prognosis in the case of elevated B7H4 expression in tumour tissues	Cell cycle	[95]
Cervical cancer	44.8–80.56%	Unclear	Inhibitory effect on immunological responses	[98,99,101,102,105]
Ovarian cancer	94.5%	Unfavourable prognosis in the case of elevated B7H4 expression in tumour tissues	Stimulation of the expression of proteins that enhance the growth and migration of cancer cells	[109,111,114,118]
Endometrial cancer	71.5%	Unclear	Unclear	[126,127,130]
Head and neck malignancies	Salivary gland carcinomas (SGC) = 50% Adenoid cystic carcinoma (ACC) = 94%	Unclear	Modulation of macrophage polarisation, creation of an anti-inflammatory microenvironment	[131,132,133]
Lung cancer	Increase	Unfavourable prognosis in the case of elevated B7H4 expression in tumour tissues	Inhibitory effect on immunological responses	[136,137,140]
Small cell lung carcinoma	2.6–6.8%	Unclear	Unclear	[138,139,140]
Lung adenocarcinoma	44.9%	Unclear	Unclear	[141,142,143]
Central nervous system malignancies	Glioma = 54.1%	Unfavourable prognosis in the case of elevated B7H4 expression in tumour tissues	Decreased expression of checkpoint genes	[145,146,147]

**Table 2:** Influence of B7H4 expression on immunological landscape in tumours. NA = not applicable.

Type of Tumour	B7H4 Expression and Immune Infiltration	References
Breast cancer	Increase in regulatory lymphocytes Decrease in cytotoxic lymphocytes	[34]
Oesophageal cancer	Increase in Foxp3+T lymphocytes, CD68+ macrophages Decrease in TIL CD3+, CD8+	[20,35,37]
Gastric cancer	Increase in Foxp3+ Tregs, macrophages, neutrophils Decrease in CD8+ lymphocytes	[40,45]
Cholangiocarcinoma	Increase in NA Decrease in CD8+ lymphocytes in the tumour stroma	[61]
Gallbladder cancer	Increase in NA Decrease in CD8+ TILs in GBC	[64]
Colorectal cancer	Increase in T CD3+, TAM Decrease in IL-9, IL-18, T CD8+	[6,75,79,82]
Renal cell carcinoma	Increase in peripheral neutrophils Decrease in NA	[87]
Bladder cancer	Increase in NA Decrease in cytotoxic lymphocytes	[92]
Cervical cancer	Increase in Tregs, CD25+FOXP3+T cells, IL-10 and TGF-β1 Decrease in T CD8+, IFN-γ, IL-2	[100,101,103,105,107]
Ovarian cancer	Increase in increased density of stromal immune infiltrations, CD11c+HLA-DRhigh APCs, IL6, IL-10, TGFB1, IFNG mRNA expression, ACKR2 mRNA, CCL2, CCL4, CCL5, CCL8, CXCL17, CXCL10, CXCL11, CXCL17 Decrease in NA	[111,113,114]
Endometrial cancer	Increase in resting memory CD4+ T cells Decrease in CD8+ cells, activated memory CD4+ T cells	[126,129,130]
Lung cancer	Increase in NA Decrease in CD8+ T	[137]
Glioma	Increase in Tregs Decrease in TILs, TAMs	[145,146]

**Table 3:** Clinical therapeutic strategies targeting B7H4.

Target	Drug	Cancer Type	Phase	NTC Number	Estimated Primary Completion
B7H4	ADC:XMT-1660	Breast, ovarian, endometrial cancer	I	NCT05377996	2026-12
B7H4	ADC:SGN-B7H4V	Solid tumours	I	NCT05194072	2025-06-30
B7H4, TOP1	ADC:AZD8205	Solid tumours	I, II	NCT05123482	2025-06-30
B7H4, PD-1	ADC:BG-C9074 and Tislelizumab	Solid tumours	I	NCT06233942	2027-09-28
B7H4, PD-1	FPA150 and Pembrolizumab	Solid tumours	I	NCT03514121	2024-03-14-results submitted, yet NA
B7H4 and CD3	bispecific antibody GEN1047	Solid tumours	II	NCT05180474	2026-01-31
4-1BB and B7H4	bispecific antibody ABL103	Solid tumours	I	NCT06126666	2024-11-15
B7H4 and CD3	bispecific antibody PF-07260437	Breast, ovarian, endometrial cancer	I	NCT05067972	2023-10-17 Terminated
